# Crystal structure of AlPCl_8_

**DOI:** 10.1107/S2056989024010661

**Published:** 2024-11-08

**Authors:** Hyeonjin Seo, Seungyong Shin, Seung-Tae Hong

**Affiliations:** a Daegu Gyeongbuk Institute of Science and Technology (DGIST), Daegu 42988, Republic of Korea; b Department of Chemistry and Chemical Biology, University of New Mexico Albuquerque New Mexico 87131 USA; c NexeriaTek Inc., Daejeon 34016, Republic of Korea; Harvard University, USA

**Keywords:** crystal structure, aluminium phospho­rus chloride, aluminium(III) chloride, phospho­rus(V) chloride, single-crystal

## Abstract

The crystal structure of aluminium phospho­rus chloride (systematic name: phospho­rus tetra­chloride tetra­chloridoa­luminate), (PCl_4_)[AlCl_4_] or AlPCl_8_, was determined and refined using single-crystal X-ray diffraction data. The compound crystallizes in the ortho­rhom­bic space group *Pbcm*. The asymmetric unit comprises one Al atom, one P atom, and five Cl atoms. The structure is characterized by isolated AlCl_4_ and PCl_4_ tetra­hedra, isostructural with FePCl_8_ and GaPCl_8_.

## Abstract 2

The crystal structure of aluminium phospho­rus chloride was determined and refined using single-crystal X-ray diffraction data. The compound crystallizes in the ortho­rhom­bic space group *Pbcm* with the asymmetric unit comprises one Al atom, one P atom, and five Cl atoms. The structure is characterized by isolated AlCl_4_ and PCl_4_ tetra­hedra, isostructural with FePCl_8_ and GaPCl_8_.

## 1. Chemical context

During our exploratory synthesis in the Mg–Al–P–Cl system, aimed at discovering new magnesium-ion conductors, we initially observed the AlPCl_8_ phase. Magnesium-ion conductors, such as MgAl_2_Cl_8_, exhibit Mg-ion conductivity of approximately 10^−7^ S cm^−1^ at 400 K (Tomita *et al.*, 2021 ▸). To enhance this ionic conductivity, we introduced an aliovalent substitution of Al with P to create magnesium-ion vacancies within the structure, following the general formula Mg_1–*x*_Al_2–*x*_P_*x*_Cl_8_.

Across a wide range of *x* values (0.1 to 1), we identified a new phase through powder X-ray diffraction (XRD) patterns, which differed significantly from that of MgAl_2_Cl_8_. Subsequent analysis revealed that this new phase matched the XRD pattern of AlPCl_8_ (Fischer & Jübermann, 1938 ▸). Since the crystal structure of AlPCl_8_ was previously unknown, we proceeded to grow single crystals without Mg to determine its structure. The resulting analysis confirmed that its crystal structure is isostructural with FePCl_8_ (Kistenmacher & Stucky, 1968 ▸) and GaPCl_8_ (Weigand *et al.*, 2009 ▸).

## 2. Structural commentary

**Figure 1:**
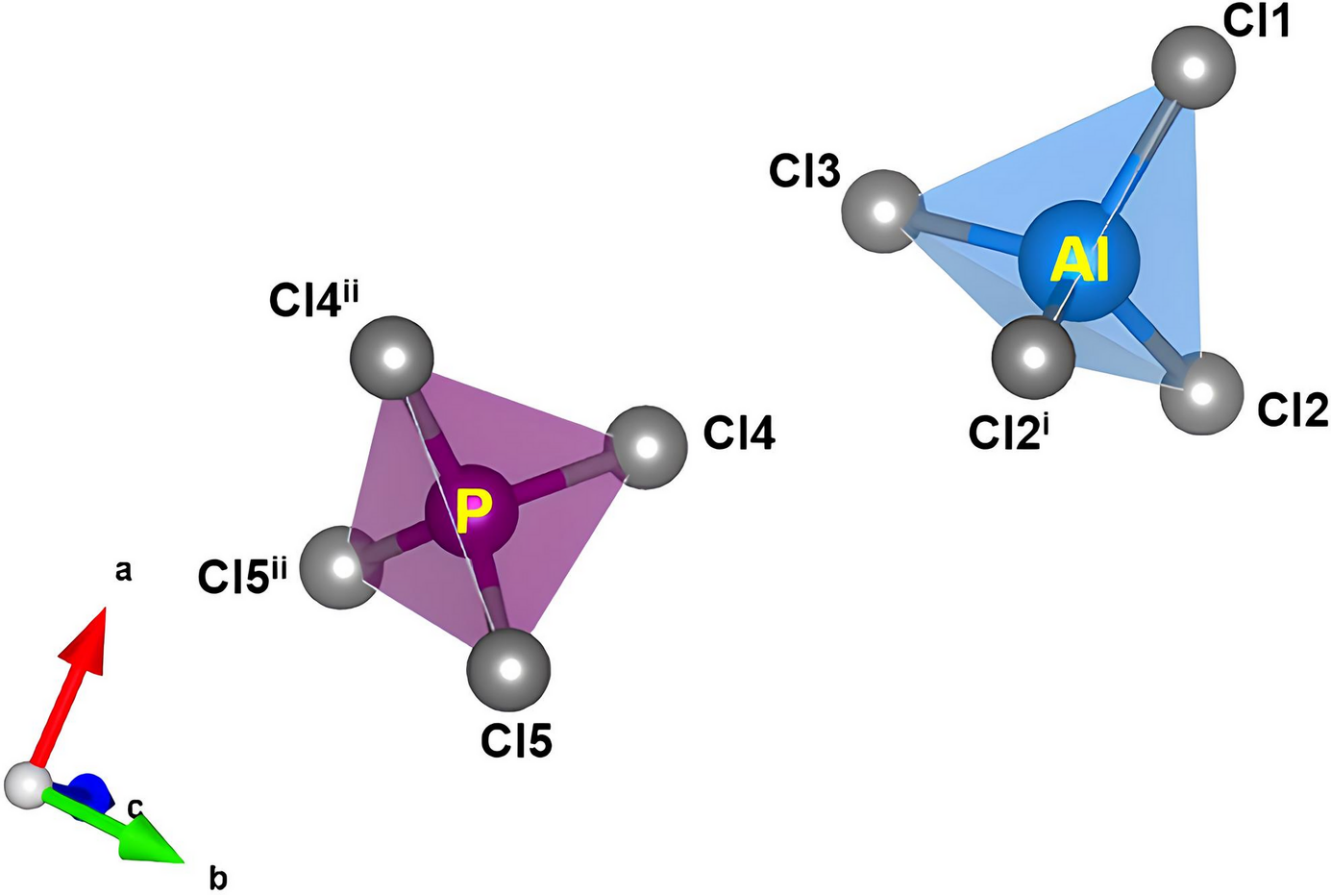
The local environments of the AlCl_4_ (blue) and PCl_4_ tetra­hedra (purple) are shown. Symmetry codes correspond to those in Table 1 ▸.

**Figure 2:**
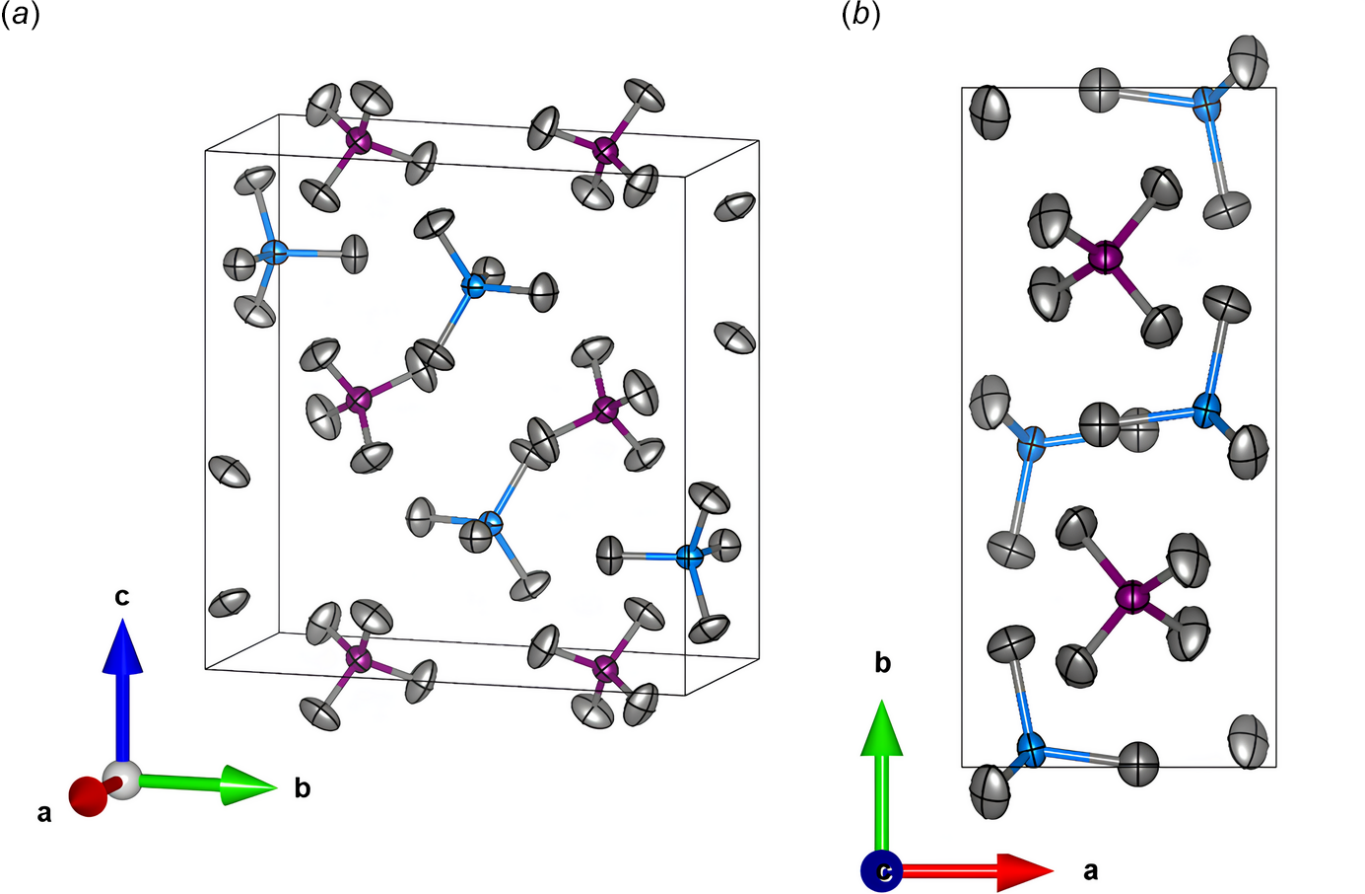
The displacement of ellipsoids of AlPCl_8_ drawn at the 50% probability level viewed from two different orientations: (*a*) approximately along the [111] direction and (*b*) along the [001] direction. The AlCl_4_ tetra­hedra are represented in blue, and the PCl_4_ tetra­hedra in purple.

Anhydrous aluminium phospho­rus chloride (AlPCl_8_) crystallizes in the ortho­rhom­bic space group *Pbcm* (Fig. 1 ▸), with a structure consisting of isolated AlCl_4_ and PCl_4_ tetra­hedra, and one Al, one P, and five Cl sites in the asymmetric unit. Al^3+^ is tetra­hedrally coordinated by four Cl atoms, with an average Al—Cl bond distance of 2.127 (2) Å, while P^5+^ is similarly coordinated, but a shorter average P—Cl bond distance of 1.899 (2) Å. These bond lengths (Table 1 ▸) are consistent with the sums of the ionic radii for Al, P, and Cl (Shannon, 1976 ▸). The local environment of each tetra­hedron is shown in Fig. 2 ▸. The crystal structure was determined to be isostructural with (FeCl_4_)(PCl_4_) (Kistenmacher & Stucky, 1968 ▸).

To validate the refined crystal structure, bond-valence sums (BVSs) were calculated using the *softBV* (Chen *et al.*, 2019 ▸) program (V1.3.1). The calculated BVS values closely match the expected ionic charges, further supporting the reliability of the structural model: Al 3.04, P 5.05, Cl1 − 0.77, Cl2 − 0.78, Cl3 − 0.78, Cl4 − 1.25, and Cl5 − 1.24.

## 3. Synthesis and crystallization

Anhydrous aluminium chloride (AlCl_3_, Alfa Aesar, anhydrous, reagent grade) and phospho­rus(V) chloride (PCl_5_, Sigma-Aldrich, 95%) were used in the experiment. A stoichiometric mixture of AlCl_3_ and PCl_5_ was ground using a mortar and pestle and then pressed into a pellet. The pellet was placed in a dry fused-silica ampoule, which was sealed under vacuum and heated in a furnace. The temperature was increased from 303 K to 573 K at a rate of 5 K min^−1^, then gradually lowered to 373 K at a rate of 0.0694 K min^−1^. The sample was then allowed to cool naturally to room temperature. Single crystals were collected at 293 K using an optical microscope in a dry room with a dew point of 223 K. A crystal, approximately 0.1 mm in size, was placed into a 0.5 mm diameter glass capillary and sealed with capillary wax (Hampton Research). The same sample was subsequently used for powder analysis.

## 4. Refinement

Details of the data collection and structure refinement are summarized in Table 2 ▸. Single-crystal X-ray diffraction data for AlPCl_8_ were collected and processed using *APEX2* (Bruker, 2006 ▸), with absorption corrections applied through *SAINT* (Bruker, 2006 ▸). The structure was solved using *SUPERFLIP* (Palatinus & Chapuis, 2007 ▸) and refined using *CRYSTALS* (Betteridge *et al.*, 2003 ▸). Three-dimensional Fourier electron-density maps were visualized using *MCE* (Rohlíček & Hušák, 2007 ▸), and structural visualizations were generated using *VESTA* (Momma & Izumi, 2011 ▸).

**Figure 3:**
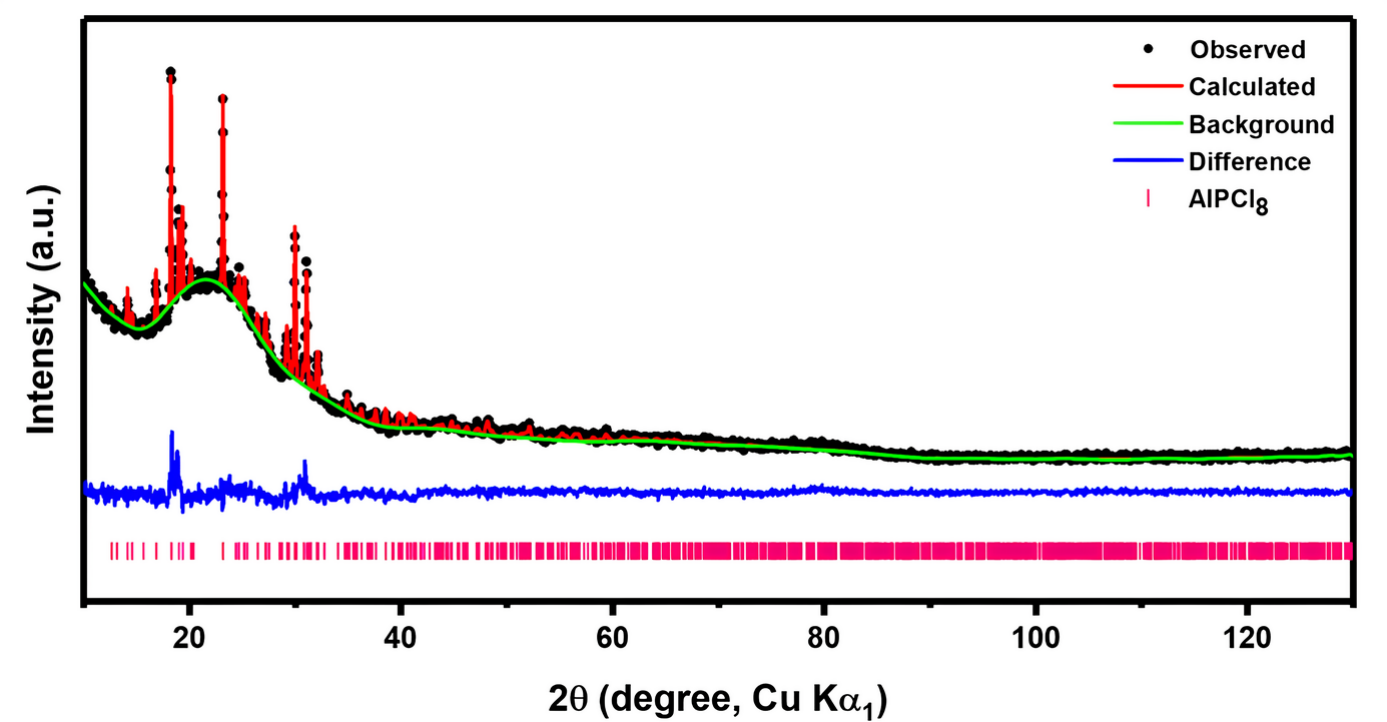
Powder X-ray Rietveld refinement profile of AlPCl_8_. Black dots indicate the observed pattern, the red line represents the calculated pattern, the blue line shows the difference between the observed and calculated patterns, and the pink tick marks correspond to the Bragg reflections positions.

The structure of AlPCl_8_ was further confirmed using the powder X-ray Rietveld refinement technique. Data were collected with a Bruker AXS D8 Advance powder X-ray diffractometer, equipped with Cu *K*α_1_ radiation in Debye-Scherrer geometry, a focusing primary Ge (111) monochromator, and a Vantec position-sensitive detector with a 6° detector slit. The powder sample was homogeneously mixed with carbon (Super C, TIMCAL) at a 1:1 weight ratio to reduce preferred orientation effects, lower effective packing density, and mitigate absorption effects. The sample was placed in a 0.5 mm glass capillary and sealed with wax to prevent air exposure. Measurements were taken over an angular range of 10° ≤ 2θ ≤ 130°, with a step size of 0.016693°, conducted over 13 h at room temperature. Powder profile refinement was performed using *GSAS-II* software (Toby & Von Dreele, 2013 ▸). The final Rietveld plot is shown in Fig. 3 ▸.

## Supplementary Material

Crystal structure: contains datablock(s) global, New_Global_Publ_Block, I. DOI: 10.1107/S2056989024010661/oi2012sup1.cif

Structure factors: contains datablock(s) I. DOI: 10.1107/S2056989024010661/oi2012Isup2.hkl

CCDC reference: 2400746

Additional supporting information:  crystallographic information; 3D view; checkCIF report

## Figures and Tables

**Table 1:** Selected geometric parameters (Å, °)

Al1—Cl2^i^	2.1223 (12)	P1—Cl5^ii^	1.9018 (12)
Al1—Cl1	2.1343 (16)	P1—Cl4^ii^	1.8959 (12)
Al1—Cl2	2.1223 (12)	P1—Cl4	1.8959 (12)
Al1—Cl3	2.128 (2)	P1—Cl5	1.9018 (12)
			
Cl2^i^—Al1—Cl1	108.79 (6)	Cl5^ii^—P1—Cl4^ii^	109.31 (6)
Cl2^i^—Al1—Cl2	112.83 (10)	Cl5^ii^—P1—Cl4	110.49 (6)
Cl1—Al1—Cl2	108.79 (6)	Cl4^ii^—P1—Cl4	108.26 (9)
Cl2^i^—Al1—Cl3	108.77 (6)	Cl5^ii^—P1—Cl5	108.97 (8)
Cl1—Al1—Cl3	108.82 (8)	Cl4^ii^—P1—Cl5	110.49 (6)
Cl2—Al1—Cl3	108.77 (6)	Cl4—P1—Cl5	109.31 (6)

Symmetry codes: (i) 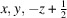; (ii) 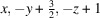.

**Table 2:** Experimental details

Crystal data	
Chemical formula	PCl_4_^+^·AlCl_4_^−^
*M*_r_	341.55
Crystal system, space group	Orthorhombic, *Pbcm*
Temperature (K)	293
*a*, *b*, *c* (Å)	6.2653 (6), 13.5033 (12), 14.0112 (13)
*V* (Å^3^)	1185.38 (19)
*Z*	4
Radiation type	Mo *K*α
μ (mm^−1^)	2.05
Crystal size (mm)	0.2 × 0.2 × 0.2

Data collection	
Diffractometer	Bruker D8 Venture
Absorption correction	Multi-scan (*SADABS*; Krause *et al.*, 2015 ▸)
*T*_min_, *T*_max_	0.664, 0.671
No. of measured, independent and observed [*I* > 2.0σ(*I*)] reflections	38491, 1399, 1061
*R*_int_	0.130
(sin θ/λ)_max_ (Å^−1^)	0.647

Refinement	
*R*[*F*^2^ > 2σ(*F*^2^)], *wR*(*F*^2^), *S*	0.093, 0.056, 1.17
No. of reflections	1061
No. of parameters	51
Δρ_max_, Δρ_min_ (e Å^−3^)	0.79, −1.04

Computer programs: *APEX2* and *SAINT* (Bruker, 2006 ▸), *SUPERFLIP* (Palatinus & Chapuis, 2007 ▸), *CRYSTALS* (Betteridge *et al.*, 2003 ▸) and *VESTA* (Momma & Izumi, 2011 ▸).

## supplementary crystallographic information

### Phosphorus tetrachloride tetrachloridoaluminate Crystal data

**Table:**

PCl_4_^+^·AlCl_4_^−^	*F*(000) = 656
*M**_r_* = 341.55	*D*_x_ = 1.914 Mg m^−^^3^*D*_m_ = 1.914 Mg m^−^^3^*D*_m_ measured by not measured
Orthorhombic, *P**b**c**m*	Mo *K*α radiation, λ = 0.71073 Å
Hall symbol: -P 2c 2b	Cell parameters from 38491 reflections
*a* = 6.2653 (6) Å	θ = 3.4–27.4°
*b* = 13.5033 (12) Å	µ = 2.05 mm^−^^1^
*c* = 14.0112 (13) Å	*T* = 293 K
*V* = 1185.38 (19) Å^3^	Block, white
*Z* = 4	0.2 × 0.2 × 0.2 mm

### Phosphorus tetrachloride tetrachloridoaluminate Data collection

**Table:**

Bruker D8 Venture diffractometer	1061 reflections with *I* > 2.0σ(*I*)
Graphite monochromator	*R*_int_ = 0.130
ω/2θ scans	θ_max_ = 27.4°, θ_min_ = 3.0°
Absorption correction: multi-scan (SADABS; Krause *et al.*, 2015)	*h* = −8→8
*T*_min_ = 0.664, *T*_max_ = 0.671	*k* = −17→17
38491 measured reflections	*l* = −18→18
1399 independent reflections	

### Phosphorus tetrachloride tetrachloridoaluminate Refinement

**Table:**

Refinement on *F*^2^	0 restraints
Least-squares matrix: full	Primary atom site location: other
*R*[*F*^2^ > 2σ(*F*^2^)] = 0.093	Weighting scheme based on measured s.u.'s Method = SQRT(W) = 1/(Data *w*ith the key S*I*GMA(/*F*O/) in list 6)
*wR*(*F*^2^) = 0.056	(Δ/σ)_max_ = 0.0003
*S* = 1.17	Δρ_max_ = 0.79 e Å^−^^3^
1061 reflections	Δρ_min_ = −1.04 e Å^−^^3^
51 parameters	

### Phosphorus tetrachloride tetrachloridoaluminate Fractional atomic coordinates and isotropic or equivalent isotropic displacement parameters (Å^2^)

**Table:**

	*x*	*y*	*z*	*U*_iso_*/*U*_eq_	
Al1	0.2214 (2)	0.47461 (11)	0.2500	0.0427	
P1	0.45745 (17)	0.7500	0.5000	0.0479	
Cl1	0.55881 (16)	0.49641 (12)	0.2500	0.0638	
Cl2	0.09231 (14)	0.53762 (10)	0.37618 (9)	0.0859	
Cl3	0.1572 (3)	0.31987 (12)	0.2500	0.0839	
Cl4	0.28015 (19)	0.80367 (9)	0.40331 (8)	0.0952	
Cl5	0.63379 (17)	0.85187 (8)	0.55069 (10)	0.0984	

### Phosphorus tetrachloride tetrachloridoaluminate Atomic displacement parameters (Å^2^)

**Table:**

	*U*^11^	*U*^22^	*U*^33^	*U*^12^	*U*^13^	*U*^23^
Al1	0.0338 (6)	0.0499 (9)	0.0445 (8)	−0.0005 (6)	0.0000	0.0000
P1	0.0443 (6)	0.0493 (7)	0.0501 (7)	0.0000	0.0000	0.0041 (7)
Cl1	0.0288 (6)	0.0861 (10)	0.0764 (8)	−0.0026 (6)	0.0000	0.0000
Cl2	0.0579 (6)	0.1250 (12)	0.0746 (7)	−0.0020 (5)	0.0154 (5)	−0.0428 (8)
Cl3	0.0807 (10)	0.0571 (9)	0.1140 (13)	−0.0146 (8)	0.0000	0.0000
Cl4	0.0936 (9)	0.1129 (12)	0.0791 (9)	0.0081 (7)	−0.0211 (7)	0.0365 (7)
Cl5	0.0842 (8)	0.0780 (9)	0.1329 (12)	−0.0163 (7)	−0.0074 (6)	−0.0404 (7)

### Phosphorus tetrachloride tetrachloridoaluminate Geometric parameters (Å, º)

**Table:**

Al1—Cl2^i^	2.1223 (12)	P1—Cl5^ii^	1.9018 (12)
Al1—Cl1	2.1343 (16)	P1—Cl4^ii^	1.8959 (12)
Al1—Cl2	2.1223 (12)	P1—Cl4	1.8959 (12)
Al1—Cl3	2.128 (2)	P1—Cl5	1.9018 (12)
			
Cl2^i^—Al1—Cl1	108.79 (6)	Cl5^ii^—P1—Cl4^ii^	109.31 (6)
Cl2^i^—Al1—Cl2	112.83 (10)	Cl5^ii^—P1—Cl4	110.49 (6)
Cl1—Al1—Cl2	108.79 (6)	Cl4^ii^—P1—Cl4	108.26 (9)
Cl2^i^—Al1—Cl3	108.77 (6)	Cl5^ii^—P1—Cl5	108.97 (8)
Cl1—Al1—Cl3	108.82 (8)	Cl4^ii^—P1—Cl5	110.49 (6)
Cl2—Al1—Cl3	108.77 (6)	Cl4—P1—Cl5	109.31 (6)

Symmetry codes: (i) *x*, *y*, −*z*+1/2; (ii) *x*, −*y*+3/2, −*z*+1.
